# Establishment of a nomogram to predict the overall survival of patients with collecting duct renal cell carcinoma

**DOI:** 10.1007/s12672-024-01140-8

**Published:** 2024-07-04

**Authors:** Weixing Jiang, Zuowei Zou, Li Wen

**Affiliations:** 1 Department of Urology, Beijing Friendship Hospital, Capital Medical University, Beijing, China; 2 Department of Nuclear Medicine, National Cancer Center/National Clinical Research Center for Cancer/Cancer Hospital, Chinese Academy of Medical Sciences and Peking Union Medical College, Beijing, 100021 China; 3 Department of Urology, National Cancer Center/National Clinical Research Center for Cancer/Cancer Hospital, Chinese Academy of Medical Sciences and Peking Union Medical College, Panjiayuan Nanli 17#, Chaoyang District, Beijing, 100021 People’s Republic of China

**Keywords:** Renal cell carcinoma, Collecting duct, Nomogram, Prediction, Prognosis

## Abstract

**Background:**

Collecting duct carcinoma (CDC) is a rare histological type of renal cell carcinoma that lacks a prognostic prediction model. In this study, we developed a nomogram to predict the prognosis of CDC patients.

**Methods:**

Data for patients (n = 247) diagnosed with CDC from 2004 to 2015 were obtained from the Surveillance, Epidemiology, and End Results (SEER) database, and the patients were randomized into training (n = 165) and validation (n = 82) cohorts. Survival outcomes were evaluated by the Kaplan–Meier method. Significant variables determined by univariate and multivariate Cox regression analyses were used to construct the nomogram. C-indexes and calibration plots were applied to evaluate the performance of the nomogram.

**Results:**

CDC patients had a median overall survival (OS) of 18.0 months (95% confidence interval: 13.7–22.3); 1-year, 3-year, and 5-year OS rates were 58.7%, 34.2%, and 29.4%, respectively. Independent prognostic factors, including age at diagnosis, tumor size, tumor grade, T stage, N stage, M stage, and surgery information, were identified by multivariate analysis. The nomogram was constructed based on significant factors in the training cohort. The C-indexes were 0.769 (training cohort) and 0.767 (validation cohort). The calibration curves for survival rates showed that the predicted and observed values were consistent.

**Conclusions:**

This study constructed a nomogram to predict prognosis in patients with CDC. The nomogram performed well in predicting the 1-year, 3-year, and 5-year OS, which can help doctors actively monitor and follow up patients.

**Supplementary Information:**

The online version contains supplementary material available at 10.1007/s12672-024-01140-8.

## Introduction

Collecting duct carcinoma (CDC), which accounts for less than 3% of renal cell carcinoma (RCC) cases, is a rare pathological type characterized by an aggressive phenotype and poor prognosis [1–3]. CDC derives from the renal collecting duct epithelium. CDC has a high propensity for distant metastasis, with poor pathological differentiation and symptoms [4, 5]. Many efforts, including advances in surgical techniques, radiotherapy, chemotherapy, targeted therapy, immunotherapy, and improvements in supportive treatment, have been made to increase the overall survival (OS) of patients with CDC [4, 6–8].

Despite modest advancements in therapy, patients with CDC have a poor prognosis. According to published studies, approximately 50% of CDC patients die from the cancer within 2 years of their initial diagnosis [9, 10]. Because of the rarity of the disease and the lack of available data, there are scant prognostic models for predicting the survival outcome of CDC tumors. Indeed, only May M and colleagues have developed a risk model, which was based on 95 patients with CDC, to predict prognosis using histopathologic and clinical parameters [9]. However, a large-population study is still needed to predict the individualized survival of patients with CDC. The Surveillance, Epidemiology, and End Results (SEER) database represents 28% of the US population, and it includes stages of cancer, treatment modalities, and survival data. As the database includes large, multi-institutional cases, it may be an essential source for rare disease research, providing strong statistical power and possibly contributing to a better understanding of CDC.

In this retrospective study, we analyzed and identified the clinicopathological features and independent prognostic factors of patients with CDC based on data retrieved from the SEER database. We further developed and validated a nomogram to aid in prognostic evaluation for individual patients with CDC.

## Patients and methods

### Data collection

The data used in this study were obtained from the online, publicly available SEER database. A data agreement form was signed, and we have submitted it to the SEER administration. As this research did not include human participants or experimental animals, it was deemed exempt by the Domain-Specific Review Board.

**Fig. 1:**
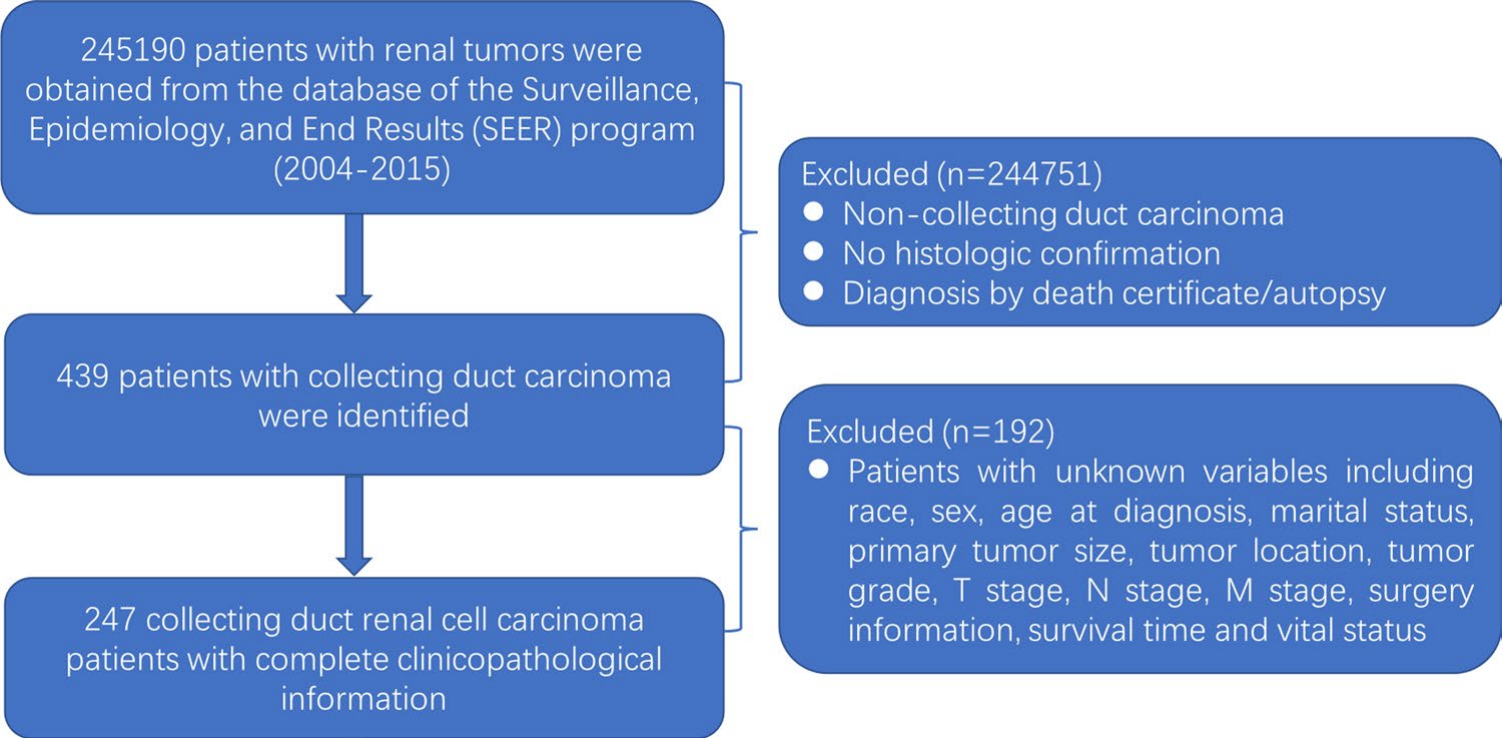
The process of screening 247 patients with collecting duct renal cell carcinoma in the SEER database for analysis

We extracted the data for this study from SEER using SEER*Stat software (Version 8.3.6). First, we identified 245,190 patients with renal tumors (International Classification of Disease for Oncology, C64) between 2004 and 2015 in the database. Then, 439 CDC patients were identified by histologic confirmation, excluding those (n = 192) with unknown variables (race, n = 5; marital status, n = 13; primary tumor size, n = 90; tumor location, n = 2; tumor grade, n = 69; T stage, n = 4; N stage, n = 1; surgery information, n = 7; and survival time, n = 1). Surgery information was identified as partial and radical nephrectomy excluding local tumor destruction, such as laser ablation (n = 2), cryosurgery (n = 2), or photodynamic therapy (n = 1). Ultimately, 247 CDC patients were eligible for analysis. The process used to generate our analytic cohort is depicted in Fig. 1.

### Data preparation and definition

The following 13 variables were obtained from the SEER program: race, sex, age at diagnosis, marital status, tumor location, primary tumor size, tumor grade, tumor-node metastasis (TNM) stage information, surgical treatment, OS in months, and vital status. “Others” of the race category included American Indian/Alaska Native or Asian/Pacific Islander. Marital status was assessed as single or married, with “single” including never married, separated/divorced, and widowed, according to SEER marital status categories. X-tile software (Version 3.6.1) was used to transform “age at diagnosis” into a categorical variable (Supplementary Fig. 1). Primary tumor size was evaluated based on the 7th AJCC staging system by categorizing as ≤ 4 cm, 4–7 cm (including 7 cm), 7–10 cm (including 10 cm) and ˃ 10 cm [11].

### Nomogram construction and validation

The enrolled CDC patients were randomized to two groups: a training cohort (n = 165) and a validation cohort (n = 82). A nomogram was developed using the training cohort. Prognostic factors were identified by univariate and multivariate Cox regression analyses, and the significant variables were used to establish the nomogram for predicting 1-, 3-, and 5-year OS rates. The established nomogram was then internally (in the training cohort) and externally (in the validation cohort) validated. The C-index was employed to evaluate the discrimination of the nomogram [12], whereby good discriminative ability was a C-index between 0.71 and 0.90; a C-index > 0.90 indicated better accuracy. The performance of the nomogram was evaluated with a calibration plot, and a plot falling on a diagonal 45° line was considered to indicate a useful nomogram.

### Statistical analysis

The final endpoints of this study were the 1-, 3-, and 5-year OS rates. Relationships between the training and validation cohorts were compared by the chi-square test or Fisher’s exact test. Survival outcomes were determined by the Kaplan–Meier method and the log-rank test. The statistical analysis was performed using the R software environment for statistical computing and graphics (version 4.0.0) with the packages rms, ggplot2, survival, and survminer. A p value less than 0.05 was considered significant.

## Results

### Patient characteristics

This study consisted of 247 CDC patients, including 169 (68.4%) males and 78 (31.6%) females. Most patients were white Americans (n = 177, 71.7%), ˃ 60 years of age (n = 126, 51.0%), and married (n = 207, 83.8%). Histologically, most tumors were grade III/IV (n = 201, 81.4%) and more than 4 cm in size (n = 180, 72.9%). Most patients had aggressive disease (T3-4 stage, n = 155, 62.7%). In addition, there was a high proportion of patients with lymph node involvement (N1 stage, n = 100, 40.5%) and metastasis (M1 stage, n = 87, 35.2%). A total of 229 (92.7%) patients underwent surgery, including partial nephrectomy (n = 58, 23.5%), total nephrectomy (n = 109, 44.1%) and cytoreductive nephrectomy (n = 62, 25.1%). Differences in variables between the training and validation cohorts were not statistically significant (p˃0.05). The clinicopathological characteristics are shown in Table 1.

**Table 1:** Patient characteristics of training and validation sets

Characteristics	Total patients	Training set (*n* = 165)	Validation set (*n* = 82)	*p* value
Race, n (%)				0.711
White Americans	177 (71.7)	120 (67.8)	57 (32.2)	
African Americans	53 (21.4)	33 (62.3)	20 (37.7)	
Others	17 (6.9)	12 (70.6)	5 (29.4)	
Sex, n (%)				0.541
Man	169 (68.4)	115 (68.0)	54 (40.0)	
Woman	78 (31.6)	50 (64.1)	28 (35.9)	
Age at diagnosis, n (%)				0.370
≤ 60	121 (49.0)	86 (71.1)	35 (28.9)	
61–70	63 (25.5)	40 (63.5)	23 (36.5)	
≥ 71	63 (25.5)	39 (61.9)	24 (38.1)	
Marital status, n (%)				0.053
Married	207 (83.8)	133 (64.3)	74 (35.7)	
Single	40 (16.2)	32 (80.0)	8 (20.0)	
Primary tumor size, n (%)				0.922
≤ 4 cm	67 (27.1)	43 (64.2)	24 (35.8)	
4–7 cm	91 (36.9)	61 (67.0)	30 (33.0)	
7–10 cm	61 (24.7)	41 (67.2)	20 (32.8)	
> 10 cm	28 (11.3)	20 (71.4)	8 (28.6)	
Tumor location, n (%)				0.301
Left	132 (53.4)	92 (69.7)	40 (30.3)	
Right	115 (46.6)	73 (63.5)	42 (36.5)	
Tumor grade, n (%)				0.101
I + II	46 (18.6)	26 (56.5)	20 (43.5)	
III + IV	201 (81.4)	139 (69.2)	62 (30.8)	
T stage, n (%)				0.754
T1	77 (31.2)	48 (62.3)	29 (37.7)	
T2	15 (6.1)	10 (66.7)	5 (33.3)	
T3	133 (53.8)	91 (68.4)	42 (31.6)	
T4	22 (8.9)	16 (72.7)	6 (27.3)	
N stage, n (%)				0.742
N0	147 (59.5)	97 (66.0)	50 (34.0)	
N1	100 (40.5)	68 (68.0)	32 (32.0)	
M stage, n (%)				0.594
M0	160 (64.8)	105 (65.6)	55 (34.4)	
M1	87 (35.2)	60 (69.0)	27 (31.0)	
Surgery, n (%)				0.304
Yes	18 (7.3)	14 (77.8)	4 (22.2)	
No	229 (92.7)	151 (65.9)	78 (34.1)	

### Clinical outcomes in patients with CDC and nomogram construction

**Fig. 2:**
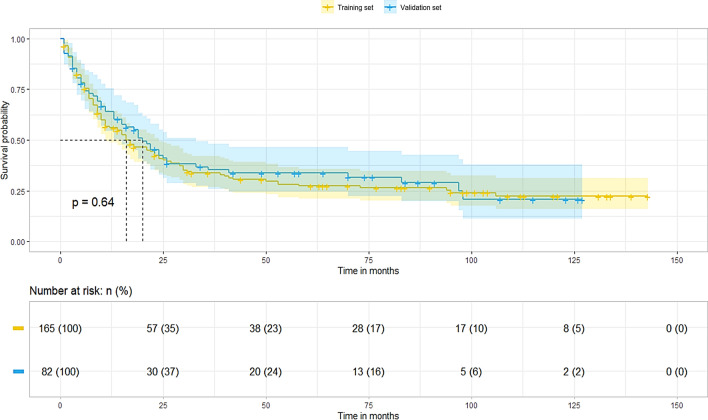
Survival analysis of patients with collecting duct renal cell carcinoma in the training and validation sets

The median overall follow-up time was 84.0 months (interquartile range [IQR]: 49.0–115.0 months), and the median OS for patients with CDC was 18.0 months (95% confidence interval CI: 13.7–22.3). Rates of 1-, 3-, and 5-year OS were 58.7%, 34.2%, and 29.4%, respectively. However, the survival difference between the training and validation cohorts was not statistically significant (median, 16.0 vs. 20.0 months, p = 0.642) (Fig. 2).

The nomogram was established based on the training cohort (n = 165), and models were constructed by univariate and multivariate regression analyses (Table 2).

**Table 2:** Independent prognostic factors analyzed using the Cox proportional hazards regression model

Variables	Univariate analysis			Multivariate analysis		
	HR	95% CI	*p* value	HR	95% CI	*p* value
Race						
White Americans	1					
African Americans	0.831	0.519–1.329	0.439			
Other	1.256	0.606–2.603	0.539			
Gender						
Female	1					
Male	1.088	0.727–1.627	0.682			
Age at diagnosis						
≤ 60	1			1		
61–70	1.148	0.726–1.817	0.555	1.199	0.739–1.945	0.462
≥ 71	1.981	1.275–3.077	0.002	1.730	1.103–2.715	0.017
Marital status						
Married	1					
Unmarried	1.158	0.707–1.897	0.559			
Tumor size (cm)						
≤ 4	1			1		
4–7	2.663	1.537–4.615	< 0.001	1.571	0.881–2.799	0.126
7–10	4.430	2.454–7.996	< 0.001	2.032	1.042–4.251	0.048
> 10	4.367	2.206–8.644	< 0.001	2.228	1.179–4.213	0.014
Tumor location						
Right	1					
Left	0.725	0.502–1.047	0.086			
Tumor grade						
I + II	1			1		
III + IV	3.753	1.953–7.212	< 0.001	1.796	1.027–3.628	0.016
T stage						
T1-2	1			1		
T3-4	2.961	1.911–4.590	< 0.001	1.479	1.009–2.487	0.021
N stage						
N0	1			1		
N1	3.210	2.183–4.721	< 0.001	1.418	1.192–2.288	0.002
M stage						
M0	1			1		
M1	4.031	2.720–5.973	< 0.001	2.316	1.481–3.622	< 0.001
Surgery						
Yes	1			1		
No	5.197	2.817–9.587	< 0.001	3.101	1.572–6.117	0.001

**Fig. 3:**
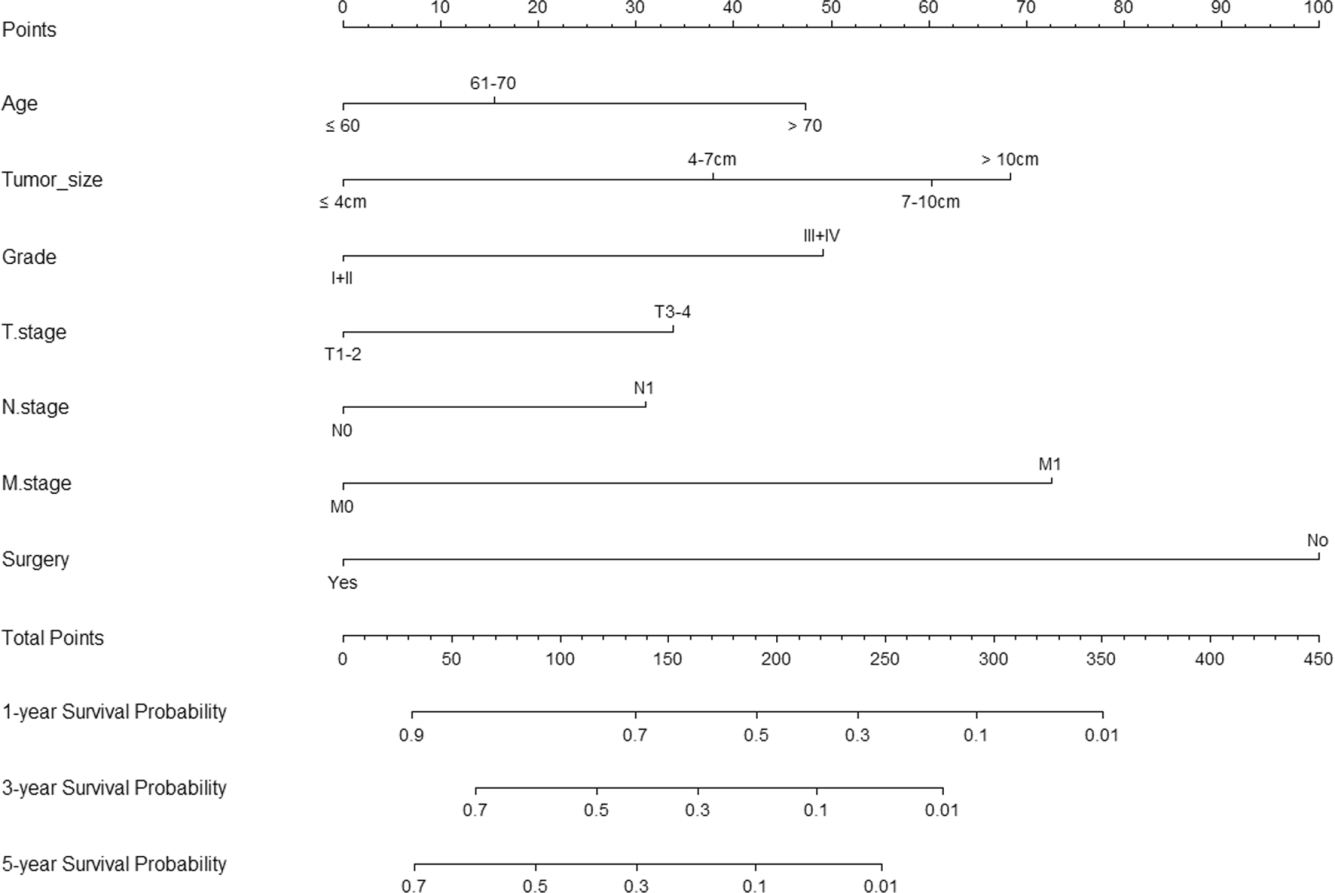
Nomogram for predicting the 1-year, 3-year and 5-year OS of patients with collecting duct renal cell carcinoma

Univariate survival analysis revealed age at diagnosis, primary tumor size, surgery, tumor grade, T stage, N stage, and M stage to be significant prognostic factors for OS, and these seven significant risk factors were examined by multivariate analysis. The results showed that age ≥ 71 (hazard ratio [HR]: 1.730, 95% confidence interval CI 1.103–2.715, p = 0.017), tumor diameter 7–10 cm (HR: 2.032, 95% CI 1.042–4.251, p = 0.048) or > 10 cm (HR: 2.228, 95% CI 1.179–4.213, p = 0.014), tumor grade III/IV (HR: 1.796, 95% CI 1.027–3.628, p = 0.016), T3/4 stage (HR: 1.479, 95% CI 1.009–2.487, p = 0.021), N1 stage (HR: 1.418, 95% CI 1.192–2.288, p = 0.002), M1 stage (HR: 2.316, 95% CI 1.481–3.662, p < 0.001), and no surgery (HR: 3.101, 95% CI 1.572–6.117, p = 0.001) were independent prognostic factors for OS. These significant factors assigned different scores according to their prognostic value were selected to construct the nomogram for predicting 1-, 3-, and 5-year OS rates (Fig. 3).

The scales of age, tumor size, tumor grade, T stage, N stage, M stage, and surgery ranged from 0 to 47.5, 0 to 68, 0 to 49, 0 to 34, 0 to 31, 0 to 72.5, and 0 to 100, respectively. Survival probability was then estimated by calculating the total score. To use the nomogram, the specific points of individual patients are located on each variable axis. The sum of these points is located on the Total Points axis, and a vertical line downward to the survival axes determines the probability of OS.

### Nomogram validation

**Fig. 4:**
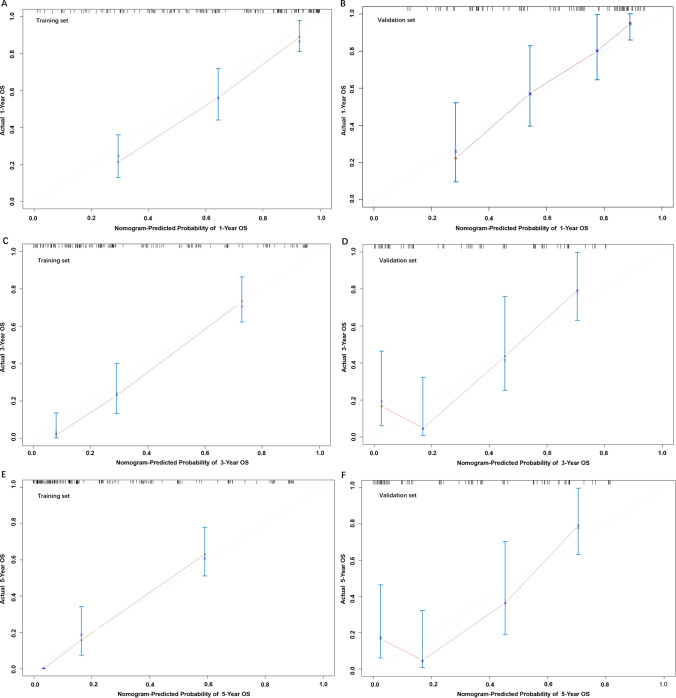
Calibration plots for predicting 1-year (**A**), 3-year (**C**), and 5-year (**E**) OS in the training set and 1-year (**B**), 3-year (**D**), and 5-year (**F**) OS in the validation set

We first evaluated the nomogram in the training set, with a C-index for OS of 0.769 (95% CI 0.697–0.841). Calibration curves of 1-, 3- and 5-year OS rates showed that the predicted and observed values of the nomogram were consistent (Fig. 4). Then, the validation cohort was used to assess the nomogram, and the OS prediction C-index was 0.767 (95% CI 0.696–0.838). Furthermore, the calibration curves of the 1-, 3- and 5-year OS rates also revealed that the predicted and observed values were consistent (Fig. 4). As illustrated in Fig. 4, the line segments in the calibration plots are close to the 45° line, which indicates good predictive ability, demonstrating that our nomogram is useful for the prediction of 1‐, 3‐, and 5‐year OS.

## Discussion

The tumor-node metastasis (TNM) staging system is the most commonly used method for predicting the survival outcome of RCC. However, patients with different histological types may have considerable survival differences, even at the same stage of RCC. CDC is a rare type of RCC with few available clinicopathological data that is associated with poor survival outcomes [3]. Nomograms have been shown to be a promising method to predict the prognosis of patients with some rare tumor types [13–15]. Thus, we sought to develop and validate a prognostic nomogram. In this study, we identified a large sample of patients with CDC from the SEER database and developed a nomogram to predict OS. The results showed that the nomogram performed well in the internal and external validation cohorts.

There are only a few published papers about CDC to date. The largest dataset is from the National Cancer Database and included 577 CDC patients [16], reporting that patients with CDC present with higher grade and stage, node positivity, and metastatic disease than those with clear cell RCC. Additionally, the OS for CDC patients is shorter than that for clear cell RCC patients (median, 13.2 vs. 122.5 months, p < 0.001). Additionally, a multicenter study including 41 CDC patients demonstrated that the main Fuhrman grades were III/IV and that 49% of CDC cases were N1-2 [5]. Moreover, 19% of CDC patients in that study had distant metastases. Another study of a population from 66 Japanese institutions consisted of 81 confirmed CDC cases, with 44.2% of patients presenting with lymph node involvement and 32.1% with metastasis [17]. The newly diagnosed CDC patients from the SEER database examined in this study also had disease with high aggressiveness. Of the 247 total CDC cases, 201 (81.4%) were grade III/IV, 155 (62.7%) T3-4 stage, 100 (40.5%) with lymph node involvement, and 87 (35.2%) with metastasis. These results are similar to those of the abovementioned reports. Furthermore, survival analysis showed that the median OS for CDC patients was 18.0 months. The rates of 1-, 3-, and 5-year OS were 58.7%, 34.2%, and 29.4%, respectively.

There is only one large study that aimed to develop a model for the prediction of the survival outcome of CDC. May M and colleagues proposed a prognostic scoring model consisting of clinical and pathological parameters based on 95 CDC patients, including the American Society of Anesthesiologists score, tumor size, Fuhrman grade, lymphovascular invasion status, and metastasis presentation. The final evaluation of the model was accurate, with a C-index value of 0.894 [9]. In our study, we used a relatively large population (n = 247) and divided these patients into training and validation cohorts. In the training cohort, 7 variables, namely, age at diagnosis, primary tumor size, surgery, tumor grade, T stage, N stage, and M stage, were associated with prognosis in univariate analysis; multivariate analysis demonstrated these factors to be independent prognostic factors for OS. Therefore, these factors were used to establish the nomogram for predicting 1-, 3-, and 5-year OS rates. Through internal self-verification in the training cohort and external verification in the validation cohort, the C-index values reached 0.769 and 0.767, respectively. The results of verification revealed that our nomogram has good predictive ability.

Research thus far has focused on predicting tumor prognosis by combining molecular markers with clinicopathological factors. The heterogeneity of CDC may also be accompanied by biological differences among patients. Overall, there is a lack of knowledge regarding the biology and molecular architecture of this tumor compared with other RCC subtypes [18]. There have been only a few reports about chromosomal aberrations, mutations, and RNA expression changes in CDCs [19–21]. Although the results of these studies have clarified the aggressiveness and rarity of CDC to some extent, no clear prognostic factors have been identified. Thus, further research is still needed.

Our study has several limitations that need to be acknowledged:

Firstly, 192 patients with CDC were excluded because of data loss. Excluding patients with missing values shrink the sample size. These may contribute to selection bias.

Secondly, the SEER database does not contain detailed information about systemic therapies, such as targeted therapy, chemotherapy, and radiotherapy. Experimental information was also not included. These may lead to model imperfection.

Thirdly, the size of the datasets used is not enough, the study was lack of external validating study.

## Conclusion

In conclusion, this study revealed that age at diagnosis, primary tumor size, surgery, tumor grade, T stage, N stage, and M stage are prognostic factors for the OS of CDC patients. Moreover, the nomogram constructed based on these factors performed well in predicting the 1-, 3-, and 5-year OS rates of CDC patients.

### Supplementary Information

Additional file 1.

Additional file 2.

## Data Availability

The datasets used and/or analyzed data in the current study are available from the corresponding author on reasonable request.
