# Supplementary material for: Establishment of a nomogram to predict the overall survival of patients with collecting duct renal cell carcinoma
Source: Discov Oncol. 2024 Jul 4;15:261. doi: 10.1007/s12672-024-01140-8 (PMC11222356; doi:10.1007/s12672-024-01140-8)
Supplement: Supplementary file 1 — Additional file 1. [file 12672_2024_1140_MOESM1_ESM.docx]

Supplementary Figure 1. Age at diagnosis was transformed into a categorical variable by grouping patients according to age: ≤ 60 years old, 61-70 years old, or ˃ 70 years old.
