# Supplementary figures and images for: Establishment of a nomogram to predict the overall survival of patients with collecting duct renal cell carcinoma
Source: Discov Oncol. 2024 Jul 4;15:261. doi: 10.1007/s12672-024-01140-8 (PMC11222356; doi:10.1007/s12672-024-01140-8)

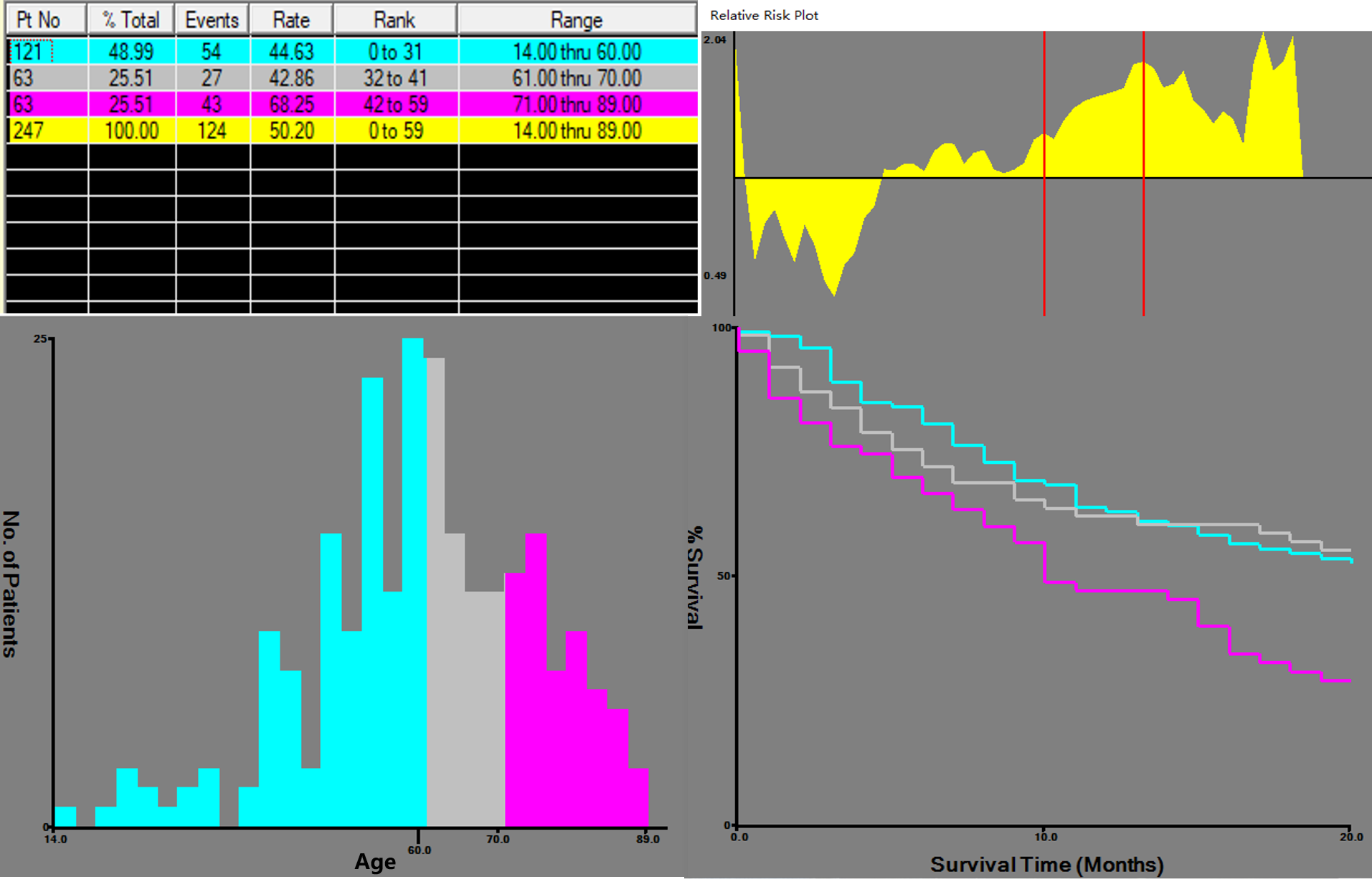

Supplement: Supplementary file 2 — Additional file 2. [file 12672_2024_1140_MOESM2_ESM.tif]
